# Midlife mental distress and risk for dementia up to 27 years later: the Nord-Trøndelag Health Study (HUNT) in linkage with a dementia registry in Norway

**DOI:** 10.1186/s12877-015-0020-5

**Published:** 2015-03-10

**Authors:** Jens Christoffer Skogen, Sverre Bergh, Robert Stewart, Ann Kristin Knudsen, Ottar Bjerkeset

**Affiliations:** 1 Division of Mental Health, Department of Public Mental Health, Norwegian Institute of Public Health, Kalfarveien 31, Bergen, 5018 Norway; 2 Alcohol and Drug Research Western Norway, Stavanger University Hospital, Stavanger, Norway; 3 Faculty of Psychology, Department of Health Promotion and Development, University of Bergen, Bergen, Norway; 4 Centre for Old Age Psychiatric Research, Innlandet Hospital Trust, Ottestad, Norway; 5 King’s College London (Institute of Psychiatry, Psychology and Neuroscience), London, UK; 6 Department of Health Registries, Norwegian Institute of Public Health, Bergen, Norway; 7 Department of Global Public Health and Primary Care, University of Bergen, Bergen, Norway; 8 Faculty of Health Sciences, Nord-Trøndelag University College (HiNT), Levanger, Norway

**Keywords:** Dementia, Midlife mental distress, Midlife risk, Anxiety, Depression

## Abstract

**Background:**

Dementia is an increasing public health challenge, and the number of individuals affected is growing rapidly. Mental disorders and symptoms of mental distress have been reported to be risk factors for dementia. The aim of this study was to examine whether midlife mental distress is a predictor for onset of dementia later in life.

**Methods:**

Using data from a large population-based study (The Nord-Trøndelag Health Study; HUNT1) linked to a dementia registry (The Health and Memory study; HMS) enabling a maximum 27 years of follow-up, we ascertained mental distress and subsequent dementia status for 30,902 individuals aged 30–60 years at baseline. In HUNT1, self-reported mental distress was assessed using the four-item Anxiety and Depression Index (ADI-4). Dementia status was ascertained from HMS, which included patient and caregiver history, cognitive testing and clinical and physical examinations from the hospitals and nursing homes serving the catchment area of HUNT1. In the main analysis, unadjusted and adjusted logistic regression models were computed for the prospective association between mental distress and dementia. In secondary analyses, two-way age and gender interactions with mental distress on later dementia were examined.

**Results:**

A 50% increased odds for dementia among HUNT1-participants reporting mental distress was found (crude odds ratio (OR): 1.52; 95% CI 1.15–2.01), and a 35% increase in the fully adjusted model (OR 1.35; 95% CI 1.01-1.80). In secondary analyses, we found evidence for a two-way interaction with age on the association between mental distress and dementia (p = 0.030): the age- and gender adjusted OR was 2.44 (95% CI 1.18–5.05) in those aged 30–44 years at baseline, and 1.24 (0.91–1.69) in 45–60 year olds.

**Conclusions:**

Our results indicate an association between midlife mental distress and increased risk of later dementia, an association that was stronger for distress measured in early compared to later midlife. Mental distress should be investigated further as a potentially modifiable risk factor for dementia.

**Electronic supplementary material:**

The online version of this article (doi:10.1186/s12877-015-0020-5) contains supplementary material, which is available to authorized users.

## Background

Dementia is caused by a number of neurodegenerative disorders, most commonly Alzheimer’s disease and vascular dementia in later life [1]. As the life expectancy of the global population increases, the number of individuals suffering from dementia is growing rapidly [2-5]. The personal and societal impacts of dementia are considerable [6-8], and it constitutes the third most common neuropsychiatric disorder in high income countries [9]. It is estimated that by 2040, about 80 million people worldwide will be affected by dementia [10] and there is a need for increased understanding of its aetiology and underlying mechanisms.

Pathology underlying dementia may be present for 10 years or more before the clinical onset [11,12], rendering it difficult to ascertain whether predictive factors in short-term follow-up studies are risk factors, or manifestations of preclinical disease [13]. Established risk factors for dementia include older age, female sex and genetic influences [14,15]. Furthermore, vascular factors have been strongly implicated for both Alzheimer’s disease and vascular dementia [14]. Other putative risk factors include smoking [16], excessive alcohol consumption [17], oxidative and inflammatory stress [15], chemical exposure [18], adiposity [19,20], and head trauma [21]. Conversely, intellectual stimulation [22,23], social activity [23], higher education and socioeconomic status [24], a healthier diet [25,26] and moderate physical activity [27] have been suggested as potential protective factors. Due to the increasing number of individuals expected to suffering from dementia and the detrimental consequences, there has been increased interest in modifiable risk and protective factors related to dementia [28,29].

Depression is frequently found to be associated with dementia, and a 2001 review concluded that clinical depression should be viewed as an independent risk factor [30]. The mechanisms explaining this association are poorly understood [31], yet it is well known that depression is also associated with cognitive dysfunction and decline [32,33]. The role of anxiety in relation to dementia is not as well-documented, although a recent study suggested a role [34]. In the general population, however, the co-morbidity of anxiety and depression is large [35,36], and many symptoms are present in both conditions, making them hard to distinguish from each other. Consequently, symptoms of depression and anxiety can be understood as indicators of general mental distress [37]. Consistent with that assumption, a study by Johansen and colleagues recently suggested a relationship between midlife mental distress and higher subsequent risk of dementia [38,39]. This study had a follow-up of more than 30 years, but was limited in that the cohort only included females and mental distress was assessed by a single question. Barnes and colleagues also found that midlife mental distress was associated with both Alzheimer’s disease and vascular dementia in a retrospective cohort study [31], although again the assessment of mental distress was primarily based on a single self-report question. Also, a study investigating mental distress in relation to the diagnosis of dementia at time of death, found that a higher score on the General Health Questionnaire (GHQ-12) was associated with increased risk of dementia mortality – although the follow-up period was more limited [40].

The aim of this study was to examine whether midlife mental distress is associated with later dementia. Specifically, a general population-based study, in which self-reported mental distress had been recorded, was linked to a dementia registry in the same catchment area, enabling individual follow-up of up to 27 years.

## Methods

### Study population

In this study, data from the population-based Nord-Trøndelag Health Study (HUNT1), carried out in 1984–86 were linked to information on dementia status from the Health and Memory Study (HMS) ascertained in 1995–2011. Both studies were conducted in the same catchment area, the Nord-Trøndelag County in Norway.

### Exposure, mental distress: the hunt1 study

The HUNT1 study was a large population-based survey of the general adult population of Nord-Trøndelag County, Norway [41,42]. HUNT1 was conducted between 1984 and 1986, and included physical examinations and self-report questionnaires on general health, health-related behaviours and socioeconomic information. Every citizen of Nord-Trøndelag County aged ≥20 years was invited to the health survey, and 77,212 people (89.4%) participated [42]. In HUNT1, attending and submitting oneself to the physical examination and answering the questionnaires was considered to be sufficient consent for participation [41]. The present study was approved by the Regional Committees for Medical and Health Research Ethics of Mid-Norway, Norway.

The population size in Nord-Trøndelag County has been relatively stable since HUNT1, and with the exception of young adults, the migration has been low [41], with a net out-migration during 1996–2000 of 0.3% per year [43].

The instrument used for assessing mental distress was the four-item Anxiety and Depression Index (ADI-4), described previously [44,45]. The instrument has been found to have reasonable performance when evaluated against the Hospital Anxiety and Depression rating scale (HADS-total score caseness (HADS-T): sensitivity 0.51; specificity = 0.93; Cohen’s ĸ = 0.55) [45]. HADS-T is a commonly used measure of mental health problems and includes 14 questions. A recent 10-year review recommended the use of HADS-T as a measure for mental distress [46].

The following four questions related to mental distress, as assessed by symptoms of anxiety and depression, were included in ADI-4:

- Calmness: “Do you by and large feel calm and good?” 1) Most of the time, 2) Often, 3) Sometimes, 4) Never.
- Nervousness: “During the last month, have you suffered from nervousness (felt irritable, anxious, tense or restless)?” 1) Never, 2) Sometimes, 3) Often, 4) Most of the time.
- Vitality: “Do you feel, for the most part, strong and fit or tired and worn out?” 1) Very strong and fit, 2) Strong and fit, 3) Quite strong and fit, 4) Neither fit nor exhausted, 5) Tired and exhausted, 6) Quite tired and exhausted, 7) Very tired and exhausted.
- Mood: “Would you say you are usually cheerful or downhearted?” 1) Very downhearted, 2) Downhearted, 3) Quite downhearted, 4) Neither cheerful nor downhearted, 5) Quite cheerful, 6) Cheerful, 7) Very cheerful.

The “nervousness” and “mood” scores were reversed before all four items were z-scored. The summed score of the items was categorised into a binary variable using the 88th percentile, as this cut-point has been shown to match the cut-off employed for HADS-Total caseness [45]. The Cronbach’s alpha in this population was 0.77, the Kaiser-Meyer-Olkin measure of sampling adequacy was 0.78, and an exploratory factor analysis indicated that the four items had satisfactory loadings on a single factor (ranging from 0.64-0.71; factor eigenvalue 1.70).

### Outcome, dementia status: the health and memory study

The primary aim of the Health and Memory Study (HMS) was to establish a dementia research registry in Nord-Trøndelag County, and included a comprehensive identification of dementia cases from hospitals and nursing homes covering Nord-Trøndelag County (see reference [47] for a detailed description). The HMS data were collected using two different procedures during the period of 1995–2011, namely: i) case note entries and ii) questionnaires and examinations. Firstly, a search of the electronic patient case notes of both hospitals serving the entire Nord-Trøndelag County was carried out to identify patients registered with a dementia diagnosis. The use of standardized dementia diagnostic procedures in these hospitals was established in 1998. Specialists in geriatric medicine and geriatric/old age psychiatry were responsible for the diagnostic procedures, and experienced geriatricians and old age psychiatrists validated the journal information retrieved for the purposes of establishing the dementia registry. The assessments of dementia status employed information regarding “both patient and caregiver history, clinical examinations, neuropsychological assessments, blood samples and imaging of the brain” ([47], p. 3). Secondly, all nursing home residents in Nord-Trøndelag were invited to participate (75.6% participation rate) in an extensive health examination with a particular focus on cognitive decline and dementia diagnosis. Using standardized interviews of the resident, the resident’s primary nurse and primary family caregiver, trained nurses assessed cognitive decline and potential presence of a dementia diagnosis.

The two data-sources; i) patients with dementia (n = 920), identified from hospital records of individuals referred to memory clinics in the two hospitals of Nord-Trøndelag County, and ii) patients with dementia (n = 620) examined in nursing homes in Nord-Trøndelag County, were combined for the purposes of this study. For a sub-group of the patients, crude information about retrospectively assessed age of onset of symptoms was also available for 306 (based on next of kin reporting retrospectively when they noticed the first symptoms of dementia). The assessment of dementia status in HMS adhered to national and international guidelines (such as ICD-10 criteria [48], the dementia with Lewy body (DLB) consortium criteria [49] and Manchester-Lund criteria [50]) and was based on patient and caregiver history, cognitive testing, and clinical and physical examinations [47]. Linkage between HUNT1 and the HMS was carried out using each participant’s personal identification number, and follow-up intervals ranged from 11 to 27 years. It has previously been established that participation rate in HUNT1 for patients diagnosed with dementia in Nord-Trøndelag County was 86%, and the mean age was 59 years at HUNT1 participation [47].

### Potential confounders: the hunt1 study

A number of covariates were considered as potential confounders. The methods and instruments have been described in detail previously [42]. In short, socio-demographic information included registry-based information on age and gender from the Norwegian Tax Administration, and self-reported marital status (“married”, “single”, “divorced”, “separated” and “widower/widow”) and education (eight categories ranging from “7-years or less of basic schooling” to “more than 4 years at the college or university”) from HUNT1. We also included on-site measurements of diastolic and systolic blood pressure, as well as the number of self-reported cardiovascular disease (CVD) indicators counting affirmative answers on one or more of the following categories: “use of anti-hypertensive medication”, “diabetes”, “heart attack”, “angina pectoris” and “stroke”. Finally we included information about health-related behaviour, including frequency of alcohol consumption over the preceding 14 days (five categories ranging from abstainer to more than 10 times), daily smoking (yes/no) and physical activity (five categories ranging from “never” to “about every day”).

### Statistical procedures

At baseline, 57,530 (74.5%) HUNT1 participants answered all of the ADI-4 items, and were eligible for further analysis. As we aimed to examine the prospective effect of mental distress prior to the development of dementia, all participants below 30 years and above 60 years at the HUNT1 participation were excluded. The reasoning behind this was that mental distress at age 60 or over could conceivably be a marker rather than a risk factor for dementia, and that risk of dementia would be negligible in persons younger than 30 years, even with more than two decades follow-up. Missing information on potential confounders ranged from 0.1% to 2.3%. This was imputed using a multivariate multiple imputation procedure with 10 imputations. All statistical analyses were performed using Stata 12.0 for Windows, except where stated otherwise [51]. First, the characteristics of the study population were described and compared between those above and below the ADI-4 cut-off using bivariate statistics (independent t-tests for continuous variables and *χ* ^2^-tests for binary and categorical variables). Unadjusted and multivariate logistic regression models were computed to investigate the association between mental distress and subsequent dementia. In secondary analyses, the two-way age and gender interaction with mental distress on later dementia status was examined. Next, as ADI-4 was a compound measure of anxiety and depression symptoms, the association between the separate items comprising the ADI-4 were investigated as exposures in a sensitivity analysis. For the participants with subsequent dementia we also estimated the mean age (years) of symptom onset, as well as the time to onset (years) from HUNT1-participation. The crude main analysis was also investigated with structural equation modeling (SEM) with “mental distress” as a continuous latent construct in Mplus version 7 [52].

Additional analyses described and compared characteristics of those with valid responses on ADI-4 and those with one or more missing covariates or outcome, as well as the predicted probability of dementia across ADI-4 percentiles (see Additional file 1). Those who had missing responses on one or more of the ADI-4 items were somewhat younger (0.5 years), were less likely to be married, had higher blood pressure, and were less likely to report 1–4 times of alcohol consumption last 14 days. They also reported less smoking, lower levels of exercise and educational attainment. There were no differences in relation to dementia status between those with and without valid responses on ADI-4.

### Ethics

The National Data Inspectorate and the Board of Research Ethics in Health Region IV of Norway approved HUNT and the current project.

## Results

The HUNT1 participants included in the current study had a mean age of 43.8 years (standard deviation (SD) 8.8) at participation and 49.9% were female. At least one dementia case was identified for all 1-year bands within the inclusion baseline age range (30 to 60 years). The participants scoring above ADI-4 cut-off were older, more often female, and less likely to be married compared to those below the cut-off (all p-values <0.001). They also had higher diastolic blood pressure, reported more CVD-indicators, less frequent alcohol consumption, more smoking, less exercise and lower educational level (p < 0.001; see Table 1). HUNT1 participants who were later registered with dementia (N = 436; 1.41%) were older and more frequently female (both p-values <0.001) than participants without registered dementia at the end of follow-up. In addition, diastolic and systolic blood pressures at HUNT1 were also higher, and CVD indicators more often reported (all p-values <0.001), as well as lower alcohol consumption (p = 0.002), less smoking (p = 0.031), and lower educational attainment (p < 0.001) in participants with later registered dementia. There were no statistically significant differences in marital status or exercise in relation to dementia status.

**Table 1:** **Description of the total sample of HUNT1 participants between 30 and 60 years who had valid responses on ADI-4 (Anxiety and Depression Index**

	**ADI-4 < 88th**	**ADI-4** ≥ **88th**	**p-value**
	**N = 28,049**	**N = 2,853**	
	**Mean (SE)/proportion (95% CI)**	**Mean (SE)/proportion (95% CI)**	
Age (years)	43.7	44.6	**<0.001**
	(0.05)	(0.17)	
Gender (% female)	48.9%	58.9%	**<0.001**
	(48.3-49.5)	(57.2-60.8)	
Marital status (% married)	83.9%	76.6%	**<0.001**
	(83.5–84.4)	(75.0–78.1)	
Diastolic blood pressure^a^	84.0	84.8	**<0.001**
	(0.06)	(0.21)	
Systolic blood pressure^a^	131.9	131.3	0.097
	(0.11)	(0.35)	
CVD indicators (% >1)^b^	8.4%	15.0%	**<0.001**
	(8.1–8.8)	(13.7–16.3)	
Alcohol consumption (% 1–4 times last 14 days)^a^	44.6%	36.1%	**<0.001**
	(44.1–45.2)	(34.3–37.8)	
Daily smoking (%)^a^	34.4%	44.5%	**<0.001**
	(33.9–35.0)	(42.6–46.3)	
Exercise (% never)^a^	9.3%	17.4%	**<0.001**
	(9.0–9.7)	(16.0–18.8)	
Higher education, (% 4-years at university-level)^a^	6.2%	4.4%	**<0.001**
	(5.9–6.5)	(3.6–5.2)	

^a^Missing values imputed using multiple imputations procedure.

^b^Based on affirmative on one or more of the following: “use of anti-hypertensive medication”, “diabetes”, “heart attack”, “angina pectoris”, “stroke”.

Bold indicates statistically significant associations (p < 0.05).

SE: Standard error.

In the crude main analyses, an increased odds for dementia among HUNT1-participants reporting mental distress (ADI-4 ≥ 88th percentile) compared to the remainder was identified (crude OR 1.52; 95% CI 1.15, 2.01; see Table 2). In the adjusted analyses, age was the principal confounding factor (age and gender adjusted OR 1.32; 95% CI 0.99, 1.75), and results were essentially the same in the fully adjusted model (adjusted for age, gender, marital status, educational attainment, blood pressure, CVD-indicators and health-related behaviours) (OR 1.35; 95% CI 1.01, 1.80). The SEM analysis yielded similar results (crude standardized path coefficient 0.057, p = 0.004).

**Table 2:** **Association between ≥88th percentile score on ADI–4 at baseline and dementia status 11–27 years after HUNT1 participation**

	**Any dementia type OR (95% CI)**	**p-value**	**Proportion of association explained by adjustment level (versus age-adjusted model)** ^**a**^
Crude (N = 436 dementia cases)	1.52 (1.15,2.01)	**0.003**	-
Age-adjusted (N = 436 dementia cases)	1.38 (1.04,1.82)	**0.027**	-
Age and gender-adjusted (N = 436 dementia cases)	1.32 (0.99,1.75)	0.055	12.8%
Fully adjusted (N = 436 dementia cases)	1.35 (1.01,1.80)	**0.043**	6.3%

N = 436 dementia cases.

^a^As computed by the following formula: $1-\frac{\mathrm{In}(adjustedOR)}{\mathrm{In}\left( age- adjustedOR\right)}$.

Bold indicates statistically significant associations (p < 0.05).

Logistic regression models. (N = 30, 902).

No statistical evidence for a gender interaction was found with mental distress on later dementia status (likelihood ratio test, p = 0.274). However, a significant two-way interaction was found between age and mental distress in relation to dementia (likelihood ratio test, p = 0.030 using age as a continuous variable). We therefore stratified for age in a post-hoc analysis, constructing two age-groups with the same range in years, age 30–44 (N = 17,843), and age 45–60 (N = 13,059). We found a stronger association between mental distress and dementia in the younger age-group (age- and gender-adjusted OR 2.44; 95% CI 1.18, 5.05), than in the older age-group (age- and gender-adjusted OR 1.24; 95% CI 0.91, 1.69). For the 306 dementia cases with available information on age of symptom onset, the 30–44 year group were also significantly younger when the symptoms first occurred (mean age 57.0 (95% CI 54.5, 59.5), compared to a mean age 73.0 (95% CI 72.2, 73.8) for the 45–60 year group, p < 0.001). There were no differences in the time from HUNT1 participation and onset (mean time to onset 18.1 years (95% CI 16.0, 20.2) in the 30–44.9 year group versus 18.2 (95% CI 17.6, 18.8) in the 45–60 year group, p = 0.919).

In sensitivity analyses, examining the predictive values of the individual ADI-4 items, three items predicted later dementia in unadjusted analyses (Nervousness, Vitality and Mood), but Nervousness was the only on remaining independent predictor in the age and gender-adjusted model (see Table 3).

**Table 3:** **Association between the individual ADI-4-items (continuous) and later dementia, unadjusted and adjusted for age and gender (N = 30, 902; n = 436 with dementia)**

**Item**	**Crude OR (95% CI)**	**Age- and gender-adjusted OR (95% CI)**
Calm	1.03 (0.94–1.14)	1.04 (0.95–1.14)
Nervous	**1.16 (1.06–1.27)**	**1.14 (1.04–1.24)**
Vitality	**1.11 (1.01–1.22)**	1.04 (0.94–1.14)
Mood	**1.20 (1.09–1.32)**	1.04 (0.94–1.15)

Bold indicates statistically significant associations (p < 0.05).

## Discussion

### Main findings

In this population-based sample followed for over two decades there was some evidence for a prospective association between mid-life mental distress and dementia, but only marginally after adjustment for potential confounders. Age explained most of the examined association, but there was little evidence for any substantial confounding from the other included covariates. A stronger association was found between mental distress and dementia for individuals younger than 45 years at baseline compared to older HUNT1 participants. To the best of our knowledge, this is the first study to investigate the association between midlife mental distress assessed in a population-based study and subsequent dementia, with a follow-up of several decades for both males and females.

### Interpretation of present findings

Given the insidious nature of dementia development [11,12], it cannot be ruled out that the associations identified in the present study are results of reverse causality (i.e. were early changes due to dementia impacts mental distress), even with a follow-up of between 11 and 27 years. There is evidence that pathology related to Alzheimer’s disease starts decades before clinical diagnosis [53,54]. In addition, although we were able to include several potentially important covariates in our analyses, residual confounding cannot be ruled out. Our findings are, however, in line with two previous studies, which both found a prospective association between midlife mental distress and dementia in women [38,39]. Also, another study found that proneness to mental distress was associated with increased risk for Alzheimer’s disease in old age [55], and a fourth study found an association between mental distress and dementia deaths [40]. However, the results from our study are not consistent with those from a retrospective cohort study where self-reported depressive symptoms in late-life were more strongly associated with increased risk of dementia than symptoms in mid-life [31]. In contrast, our findings indicate that mental distress in early mid-life is more strongly associated with an increased risk for dementia, than mental distress measured in late mid-life. Participants aged >60 years were excluded from our study, as it was envisaged to be difficult to differentiate early symptoms of dementia from symptoms of mental distress in this group.

A number of neurological and cardiovascular mechanisms could explain the observed association between mental distress and risk of dementia, including structural and functional hippocampal damage [55], and other negative effects on the hypothalamic-pituitary-adrenal axis [38,55], as well as hypertension as a potential mediator between mental distress and dementia [38]. Studies have indicated that there may be a two-way/bidirectional relationship between cardiovascular disease and depression [56,57], and this may also be true for mental distress in general. Further, midlife mental distress may be a result of increased vulnerability to stressors and dysfunctional coping strategies related to previous negative experience or genetic influences, which also constitute risk factors for dementia [39,58-60]. The finding from the sensitivity analysis that “Nervousness” was the only item independently associated with dementia after adjustment for age and gender, may suggest that this symptom of anxiety was the primary driver for dementia risk in our sample. Although this finding should be interpreted with caution, it does lend support to previous findings suggesting cortisol and exitotoxicity as a mechanism linking stress with dementia [61,62].

Associations between midlife mental distress and dementia may also be due to mental distress being a marker of rather than a risk factor for dementia [38]. Even for longer follow-up periods, there is evidence suggesting that the prodromal phase of dementia in some cases may be present several decades before dementia is manifest [54]. However, our age-stratified results, in which we found increased risk for dementia in the younger rather than the older group who reported mental distress, is not consistent with this. This may, however, be due to important differences in the characteristics of early (before 65 years of age) versus late-onset dementia (65 years or more), such as the disease progression, and symptom profile. During the past decades, it has become more common to differentiate between early- and late-onset dementia, each potentially associated with different epidemiology, genetics, clinical presentation and disease progression [63,64].

The differential association between mental distress and risk of dementia by age could potentially be related to differences in types and/or timing of onset of dementia. For example, midlife mental distress may be a more potent risk factor for earlier onset dementia. This notion may be supported by our finding that the younger group had a symptom onset on average 16 years earlier than the older group, while the time-to-dementia-onset was similar. The impact of mental distress on the development of dementia, if causal, might be affected by the duration of the distress, and as such, it may be that only prolonged mental distress has an effect on the risk of dementia [38,55]. In support of the greater impact of experiencing mental distress at younger age, early onset depressive disorder have been found to be a greater illness burden across a range of indicators compared to later onset depression [65]. Therefore, it is possible that mental distress has to be both present in early adult life and sustained into late adulthood and early old age [66]. Another explanation may be that the increased risk observed in the younger age group is a function of the interval between exposure and dementia, as the younger participants are less likely to have died during follow-up.

### Strengths and limitations

The present study has several strengths. First, it employed a linkage between a large population-based study with a high overall participation rate (92.3% for those aged 30–60 at participation) [42] and a comprehensively ascertained dementia registry in the same catchment area [47]. Also, the participation rate in HUNT1 for patients diagnosed with dementia in Nord-Trøndelag County was 86% [47], indicating that the linkage between HUNT1 and HMS can be considered valid for research purposes. Second, the linkage enabled a follow-up ranging from 11 to 27 years, decreasing the likelihood that self-reported mental distress is a marker of early stages of dementia. Third, the established dementia registry used multiple sources of information to detect and validate the dementia diagnoses, securing a high degree of certainty/reliability related to dementia diagnosis. Finally, we were able to include a range of potentially relevant confounders for the main analysis, as well as investigating the potential two-way interaction between age and gender, and mental distress on later dementia.

Some limitations are pertinent for the interpretation of the results from the present study, and should be borne in mind. First, the measure of mental distress employed in this study is not a well-validated instrument, and the psychometric properties are somewhat unclear. The range of symptoms related to mental distress that was assessed was limited, and therefore the phenomenon of mental distress as measured may be somewhat faceted and have lower specificity than validated instruments. The ADI-4, has, however, been used in several other studies and is considered as an acceptable, but crude measure of symptoms of mental distress [44,45]. Aware of this issue, we also investigated the separate items, three of which were positively associated with dementia, as well as investigating the main association using SEM-analysis with “mental distress” as a continuous latent construct. The internal consistency of the instrument was good, and an exploratory factor analysis indicated that all four items measured the same latent construct. In addition, any deficiencies in the measurement’s accuracy will have obscured rather than exaggerated associations of interest.

Second, the limited number of dementia cases decreased the precision of our estimates, restricting the ability to adjust for potential confounders in the age-stratified analyses. There were also too few cases to investigate meaningfully the impact of mid-life mental distress on different sub-types of dementia. It is, however, difficult to discern the different types of dementia conditions, and in many cases the discrimination is poor, even between Alzheimer’s and vascular dementia [38,67,68].

Third, the dementia cases which are included in the dementia registry cannot be assumed to be representative of all dementia cases residing in Nord-Trøndelag; for example, they may well be more severe [47] than the community cases not identified [69]. Also, individuals institutionalized (e.g. at mental health institutions) were not invited to participate. Although not all people suffering from dementia in Nord-Trøndelag County during the HMS study period 1995–2011 have been identified (for example those not yet diagnosed or diagnosed by other health care workers in primary care) [47], the risk estimates should only be moderately deflated, since undetected dementia cases are likely to constitute only a small fraction of the subjects considered non-cases, with misclassification unlikely to have substantial effects on observed differences in risk [47]. In addition, false positive dementia cases included in the HMS study are unlikely to have been common enough to exert an influence.

Fourth, onset of symptoms was only available for a sub-sample of the dementia cases, and was assessed in retrospect: At inclusion in the study, a next of kin was asked to report retrospectively when they noticed the first symptoms of dementia. Based on this information, the age of onset of dementia was estimated. Obviously, this information could be biased, and serves as only a crude indication for time of onset. Also, the date of diagnosis was not available, precluding any event-related analysis.

Fifth, there were few dementia cases (n = 48) among those aged under 45 years at baseline, and associations of interest in this group were thus imprecise, with wide confidence intervals. The small number of cases in the younger group also precluded any meaningful adjustment for potential confounders beyond age and gender.

Sixth, we are unable to assess and control for differential attrition, migration or mortality during follow-up, an issue which could lead to bias. There is, however, little reason to believe that these factors would lead to an inflation of the associations of interest.

Finally, there are a number of risk factors active between ascertainment of exposure and development of dementia – and these may potentially modify the association between mental distress and later dementia. The intervening risk factors could, however, reduce the likelihood of finding an association in a study with this long follow-up and controlling for subsequent factors might lead to an over-adjustment [70].

## Conclusions

In summary, we found an association between midlife mental distress and risk of later dementia, which seemed to be stronger for mental distress measured in early midlife compared to late midlife. Based on the finding of the present study, in addition to those from similar studies, it is conceivable that mental distress (i.e. anxiety and depressive symptoms) is a potentially modifiable risk factor for dementia [29,68,71,72]. Although estimating the number of dementia cases which could be attributable to mental distress is beyond the scope of the present study, there are indications that adverse psychological states during the life-course impact the risk of dementia to a great extent [68]. Future research should clarify consistency of findings for established measures of mental distress and over a longer follow-up period in order to gain further knowledge about these associations.

## Additional file

Additional file 1: Table S1. Comparison between those with valid responses on ADI-4 (75.7%) versus those with one or more missing components of ADI-4 (24.3%). N=40,803. **Figure S1.** Predicted estimated probability of dementia across ADI-4 percentiles (N=30,902).

## Abbreviation

HUNT: The Nord-Trøndelag Health Study
HMS: The Health and Memory Study
ADI-4: Four-item anxiety and depression measure
OR: Odds ratio
CVD: Cardiovascular disease
HADS-T: The Hospital Anxiety and Depression Scale, total score
DLB consortium: The dementia with Lewy body consortium
95% CI: 95% Confidence interval
