# Supplementary material for: Midlife mental distress and risk for dementia up to 27 years later: the Nord-Trøndelag Health Study (HUNT) in linkage with a dementia registry in Norway
Source: BMC Geriatr. 2015 Mar 10;15:23. doi: 10.1186/s12877-015-0020-5 (PMC4571744; doi:10.1186/s12877-015-0020-5)
Supplement: Additional file 1: Table S1. — Comparison between those with valid responses on ADI-4 (75.7%) versus those with one or more missing components of ADI-4 (24.3%). N=40,803. Figure S1. Predicted estimated probability of dementia across ADI-4 percentiles (N=30,902). [file 12877_2015_20_MOESM1_ESM.docx]

**Additional file 1: Table S1: Comparison between those with valid responses on ADI-4 (75.7%) versus those with one or more missing components of ADI-4 (24.3%). N=40,803.**

|  | Valid responses on ADI-4 (n=30,902) | Missing responses on ADI-4 (n=9,901) | p-value |
| --- | --- | --- | --- |
| Age (SE) | 43.8 (0.1) | 43.3 (0.1) | **<0.001** |
| Gender (% female) | 49.9% | 49.1% | 0.191 |
| Marital status (% married)^a^ | 83.2% | 80.1% | **<0.001** |
| Diastolic blood pressure (SE) ^a^ | 84.0 (0.1) | 84.4 (0.1) | **0.003** |
| Systolic blood pressure (SE) ^a^ | 131.8 (0.1) | 132.5 (0.2) | **0.003** |
| CVD indicators (% >1)^b^ | 9.1% | 9.4% | 0.304 |
| Alcohol consumption (% 1-4 times last 14 days) ^a^ | 43.8% | 30.0% | **<0.001** |
| Daily smoking (%) ^a^ | 35.4% | 30.4% | **<0.001** |
| Exercise (% never) ^a^ | 10.0% | 14.7% | **<0.001** |
| Higher education (% 4-years at university-level) ^a^ | 6.0% | 4.2% | **0.003** |
| Dementia (%) | 1.2% | 1.4% | 0.101 |

^a^ missing values imputed using multiple imputations procedure

^b^ Based on affirmative on one or more of the following: “use of anti-hypertensive medication”, “diabetes”, “heart attack”, “angina pectoris”, “stroke”.

Comment: Those who had missing responses on ADI-4 were somewhat younger (0.5 years), were less likely to be married, had higher blood pressure, and were less likely to report 1-4 times of alcohol consumption last 14 days. They also reported less smoking, lower levels of exercise and educational attainment. There were no differences in relation to dementia status between those with and without valid responses on ADI-4.

**Additional file 1: Figure S1: Predicted estimated probability of dementia across ADI-4 percentiles (N=30,902).**
